# Langerhans cells and SFRP2/Wnt/beta‐catenin signalling control adaptation of skin epidermis to mechanical stretching

**DOI:** 10.1111/jcmm.17111

**Published:** 2022-01-12

**Authors:** Joanna K. Ledwon, Elbert E. Vaca, Chiang C. Huang, Lauren J. Kelsey, Jennifer L. McGrath, Jacek Topczewski, Arun K. Gosain, Jolanta M. Topczewska

**Affiliations:** ^1^ Department of Surgery Plastic Surgery Division Northwestern University Feinberg School of Medicine Stanley Manne Children's Research Institute, Ann and Robert H. Lurie Children's Hospital of Chicago Chicago Illinois USA; ^2^ University of Wisconsin Joseph J Zilber School of Public Health Milwaukee Illinois USA; ^3^ Department of Pediatrics Northwestern University Feinberg School of Medicine Stanley Manne Children’s Research Institute, Ann and Robert H. Lurie Children's Hospital of Chicago Chicago Illinois USA

**Keywords:** epidermis, Langerhans cells, mechanical forces, mechanotransduction, SFRP2, skin growth, tissue expansion, Wnt signalling

## Abstract

Skin can be mechanically stimulated to grow through a clinical procedure called tissue expansion (TE). Using a porcine TE model, we determined that expansion promptly activates transcription of *SFRP2* in skin and we revealed that in the epidermis, this protein is secreted by Langerhans cells (LCs). Similar to well‐known mechanosensitive genes, the increase in *SFRP2* expression was proportional to the magnitude of tension, showing a spike at the apex of the expanded skin. This implies that *SFRP2* might be a newly discovered effector of mechanotransduction pathways. In addition, we found that acute stretching induces accumulation of b‐catenin in the nuclei of basal keratinocytes (KCs) and LCs, indicating Wnt signalling activation, followed by cell proliferation. Moreover, TE‐activated LCs proliferate and migrate into the suprabasal layer of skin, suggesting that LCs rebuild their steady network within the growing epidermis. We demonstrated that in vitro hrSFRP2 treatment on KCs inhibits Wnt/b‐catenin signalling and stimulates KC differentiation. In parallel, we observed an accumulation of KRT10 in vivo in the expanded skin, pointing to TE‐induced, SFRP2‐augmented KC maturation. Overall, our results reveal that a network of LCs delivers SFRP2 across the epidermis to fine‐tune Wnt/b‐catenin signalling to restore epidermal homeostasis disrupted by TE.

## 1 INTRODUCTION

Tissue expansion (TE) is a clinical procedure that stimulates skin growth through repetitive mechanical stretching.^1^, ^2^ Despite a long history of clinical application, the molecular mechanisms involved in skin response to TE are still largely unknown. Porcine skin is used extensively in biomedical research to study wound healing, infectious disease and radiation, because it closely resembles the human homologue.^3^ Given this, porcine became an excellent animal model to study the mechanical properties and molecular mechanisms of TE.^4^, ^5^, ^6^, ^7^, ^8^ Our previous study on genome‐wide transcriptional changes in TE revealed that mechanical forces from the expander activate pathways related to immune response, metabolism, muscle contraction and cytoskeleton organization in a time‐dependent manner.^4^ We noted *SFRP2* (Secreted frizzled‐related protein 2), a well‐known modulator of Wnt signalling, among the most upregulated genes during expansion.^4^ Here, we continue our investigation into the role of SFRP2 in skin growth and regeneration stimulated by TE.

SFRP2 is a potent signalling molecule recognized for its ability to modulate Wnt/beta‐catenin signalling. It belongs to a family of proteins that can bind directly to Wnt ligands to prevent receptor binding and activation of Wnt signalling.^9^ SFRP2 has multiple biological roles in diverse cellular processes, including tissue development^10^ and tissue homeostasis.^11^ It has previously been reported that SFRP2 is involved in proliferation and energy metabolism in cardiac fibroblasts^11^ and regulates cell proliferation, survival and a superior regenerative phenotype of mesenchymal stem cells.^12^, ^13^ In hyperpigmented skin, such as melasma, solar lentigo and acutely UV‐irradiated skin, increased expression of SFRP2 resulted in enhanced melanogenesis through positive regulation of Wnt/beta‐catenin signalling.^14^ An agonistic function of SFRP2 was also shown as its synergy with Wnt16b increased cancer cell proliferation, migration and drug resistance.^15^ Together, the literature indicates that SFRP2 modulates Wnt signalling and contributes to skin development and homeostasis, but it has not previously been implicated in skin mechanotransduction.

Langerhans cells (LCs), dendritic cells of the epidermis, are involved in maintaining epidermal health and tolerance to commensal microorganisms, and in adaptive immune response to pathogens invading the epidermis.^16^, ^17^ LCs originate from embryonic precursors, mainly liver monocytes, and are recruited to the epidermis during development. Of note, LCs are also capable of local self‐renewal, as shown in wound healing, inflammation and homeostatic conditions.^18^, ^19^ LC characteristics are very similar between different species, including human, porcine and mouse^20^ and CD207/Langerin is a popular marker, also used in this study, to identify epidermal LCs.^21^

Here, we describe *SFRP2* as a new target gene of mechanotransduction which modulates Wnt/beta‐catenin signalling and the behaviour of keratinocytes (KCs). We show that secretion of SFRP2 by LCs plays a key role in maintaining epidermal homeostasis. While a typical TE procedure takes several weeks before reconstructive surgery, we learned that stretching activates regenerative mechanisms within hours.

## 2 MATERIALS AND METHODS

### 2.1 Porcine tissue expansion models

Animals used in this study were treated as approved by protocol from Northwestern University Institutional Animal Care and Use Committee (IACUC# LCH14‐006), and NIH standards provided in the ‘Guide for the Care and Use of Laboratory Animals’. We have performed eight protocols of tissue expansion (TE) using hairless 5–6 weeks old females Yucatan minipigs,^22^ which were selected due to a minimal skin pigmentation. The rectangular tissue expanders (PMT Corp.), commonly used in the clinical settings, were placed anteriorly over the ribs (TE models: Ex‐1h, Ex‐3d, Ex‐10d^(7+3)*^, Ex‐10d^(7+3)^) or posteriorly over the abdomen (TE models: Ex‐24h, Ex‐7d, Ex‐14d^(7+7)*^, Ex‐14d^(7+7)^) (Figure S1a,b). The ‘*’ indicates the models with a double 30 ml injection. The expanders were inflated once with 60 ml of saline for 1 hour, 24 hours, 3 days or 7 days prior to euthanasia, or twice of either 30 ml or 60 ml at 10 and 3 days or 14 and 7 days prior to euthanasia, per approved protocol. The three skin biopsies for RNA study and one for histological evaluation were collected in each category: the apex (‘a’), the middle (‘b’) and the periphery (‘d’) of the expanded skin immediately prior to euthanasia (Figure S1c). The biopsies from the similar locations on the contralateral sites were used as controls.

### 2.2 RNA isolation

The full‐thickness skin punch biopsies were preserved in All Protect Tissue Reagent (Qiagen) according to manufacturer's recommendations, and RNA was extracted as described previously.^4^ Briefly, tissue was homogenized using the 3 mm zirconium beads (Benchmark Scientific) and BeadBug homogenizer. Total RNA was isolated using RNAeasy Mini Kit (Qiagen) followed by DNAse I digestion. The quality and quantity of RNA were evaluated by NanoDrop (Thermo Fisher), the agarose gel electrophoresis and fluorometric method (Qubit^TM^).

### 2.3 Real‐time PCR analysis

Synthesis of cDNA was performed using the High‐capacity cDNA Reverse Transcription kit (Life Technologies) and 200 ng of total RNA according to the manufacturer's recommendations. The Real‐time PCR analysis (qRT‐PCR) was performed on StepOnePlus Real‐Time PCR system (Applied Biosystems) using TaqMan Assay (Applied Biosystems) in a 10 μl reaction mixture per manufacturer recommendations. Cycling conditions included an initial denaturation at 95°C for 15 min, then 45 cycles at 95°C for 15 s and 60°C for 1 min. The cycle quantification values were calculated using the threshold cycle method. Fold change of gene expression was calculated with the $2^{‐\Delta \Delta C_{T}}$ method.^23^ For the animal studies, the gene expression results were normalized to *B2M* expression and for in vitro experiments with human cells results were normalized to the normalization factor, which was calculated as a geometric mean of three reference genes: *GAPDH*, *HPRT* and *HMBS1*.

### 2.4 Histology

The tissue biopsies were fixed in 10% neutral buffer formalin for 24 hours at room temperature. The post‐fixation processing, performed with Spin Tissue Processor (Thermo Fisher), included 1 hour fixation in 10% neutral buffer formalin, 30 min incubation in Pen‐Fix (Richard‐Allan Scientific), dehydration in ethanol series (1 × 80%, 2 × 95%, 2 × 100%) 30 min each, then incubation in two changes of xylene for 1 hour each, and in three changes of paraffin for 60 min each. Samples were embedded using paraffin embedding centre and cut at 5 μm using Microtome (EC350‐2, Thermo Fisher). The sections were mounted on Superfrost Plus Microscope Slides (Thermo Fisher) using Eco‐Mount medium (Biocare Medical) and evaluated by haematoxylin and eosin.

### 2.5 Immunofluorescence and immunohistochemistry staining

Immunofluorescence staining (IF) and immunohistochemistry (IHC) staining was performed on paraffin sections deparaffinized in CitriSolv Hybrid solution (Decon) and gradually rehydrated. Permeabilization was done with 0.1% Tween‐20 or 0.05% Triton X‐100 in TBS (TBST). For antigen retrieval, the Target Retrieval Solution (Dako) or Tris‐EDTA buffer (10 mM Tris base, 1 mM EDTA solution, 0.05% Tween‐20, pH 9.0) for KRT10 was used at 95°C for 20–30 min, then cooled at room temperature for 30 min and washed in PBS and PBST. Blocking was done with 10% normal serum in PBS at room temperature for 1.5 h. The sections were incubated overnight at 4°C with the primary antibodies: anti‐SFRP2 (1:500, C2C3, Genetex) anti‐CD207 (1:100, Novus), anti‐beta‐catenin (1:500, Thermo Fisher), anti‐KRT10 (1:100, RKSE60, Thermo Fisher) or 1 hour at room temperature with anti‐KRT5 (1:50, RCK102, Thermo Fisher). For IF of CD207, the biotin‐SP AffiniPure donkey anti‐rat IgG antibody (1:500, Jackson ImmunoResearch) and Vectastain Elite ABC kit (Vector Labs) were used according to manufacturer recommendations. Following secondary antibodies were used: goat anti‐mouse IgG Alexa Fluor Plus 488 (1:500, Thermo Fisher), donkey anti‐rabbit Alexa Fluor Plus 488 (1:500, Thermo Fisher), Cy‐3‐conjugated Streptavidin (1:1000), goat anti‐rabbit IgG HRP (1:500, Invitrogen) for 1 hour at room temperature. Nuclei were counterstained using DAPI in SlowFade Gold antifade reagent (Invitrogen). To reduce auto‐fluorescence Autofluorescence Reducing Reagent was used (MaxVision). The IF/IHC staining of SFRP2, SFRP2/CD207 and Ki‐67 was performed by Histology Core at Northwestern University. Staining without the primary antibodies was performed as a negative control.

### 2.6 Fluorescence microscopy

The slides were imaged on a Zeiss LSM880 confocal microscope using 40x or 63x oil objective. The exposure time was identical between controls and experimental slides. The Blue Zen software was used to measure fluorescence intensity.

### 2.7 Beta‐catenin fluorescence signal quantification

The accumulation and distribution of beta‐catenin in individual cells were calculated based on Alexa 488 fluorescence intensity using Blue Zen software (Zeiss). The numerical values were extracted from each histogram attached to the collected image. The optical sections were selected based on similar intensity of DAPI, and the mean value of signal intensity (sum of pixels intensity) was calculated from at least four sections, from each Z‐stack. Minimum six sections were tested for each tissue sample.

### 2.8 HaCaT in vitro culture

The cells were purchased from AddexBio and cultured in 5% CO_2_ at 37°C in DMEM GlutaMax medium, supplemented with 10% FBS (Gibco), sodium pyruvate and high glucose. Cells were synchronized in cell cycle by serum starvation for 16 hours, grown to 60% or 100% confluency, and then treated with recombinant protein hrSFRP2 (R&D system) at concentration 20 nM or 40 nM for 6 h. Untreated cells were used as control. Each experiment was performed in triplicates, and collected cells were used for qRT‐PCR analysis and cell cycle assay.

### 2.9 Cell cycle analysis

Cell cycle analysis was performed using Propidium Iodide Flow Cytometry Kit (Abcam) according to manufacturer's protocol. Briefly, 5x10^5^ cells in single cell suspension were fixed in 66% ethanol, then rehydrated in PBS and stained with Propidium iodide for 30 min at 37°C in the dark with presence of RNaseA. The signal intensity was measured on flow cytometer (BD FacsAria SORP 5‐Laser, Biosciences) at 488 nm laser illumination and analysed at Northwestern University Flow Cytometry Core Facility.

### 2.10 Quantitative analysis of Langerhans cells number

At least 10 microscopic fields per each section (n ≥ 3) were photographed and analysed; the number of LCs was assessed based on expression of CD207 (Novus).

### 2.11 Normal human skin sections

The normal human skin tissue was purchased from the Northwestern University Mouse Histology and Phenotyping Laboratory.

### 2.12 Statistics

All statistics were performed using GraphPad Prism 7 software (GraphPad Software, Inc.). The significance of obtained results was calculated with unpaired Student t‐test. All experimental results are presented as mean ±SD. *p* values ≤0.05 were considered as significant.

## 3 RESULTS

### 3.1 Transcriptional activation of *SFRP2* correlates with a magnitude of expansion

**FIGURE 1:**
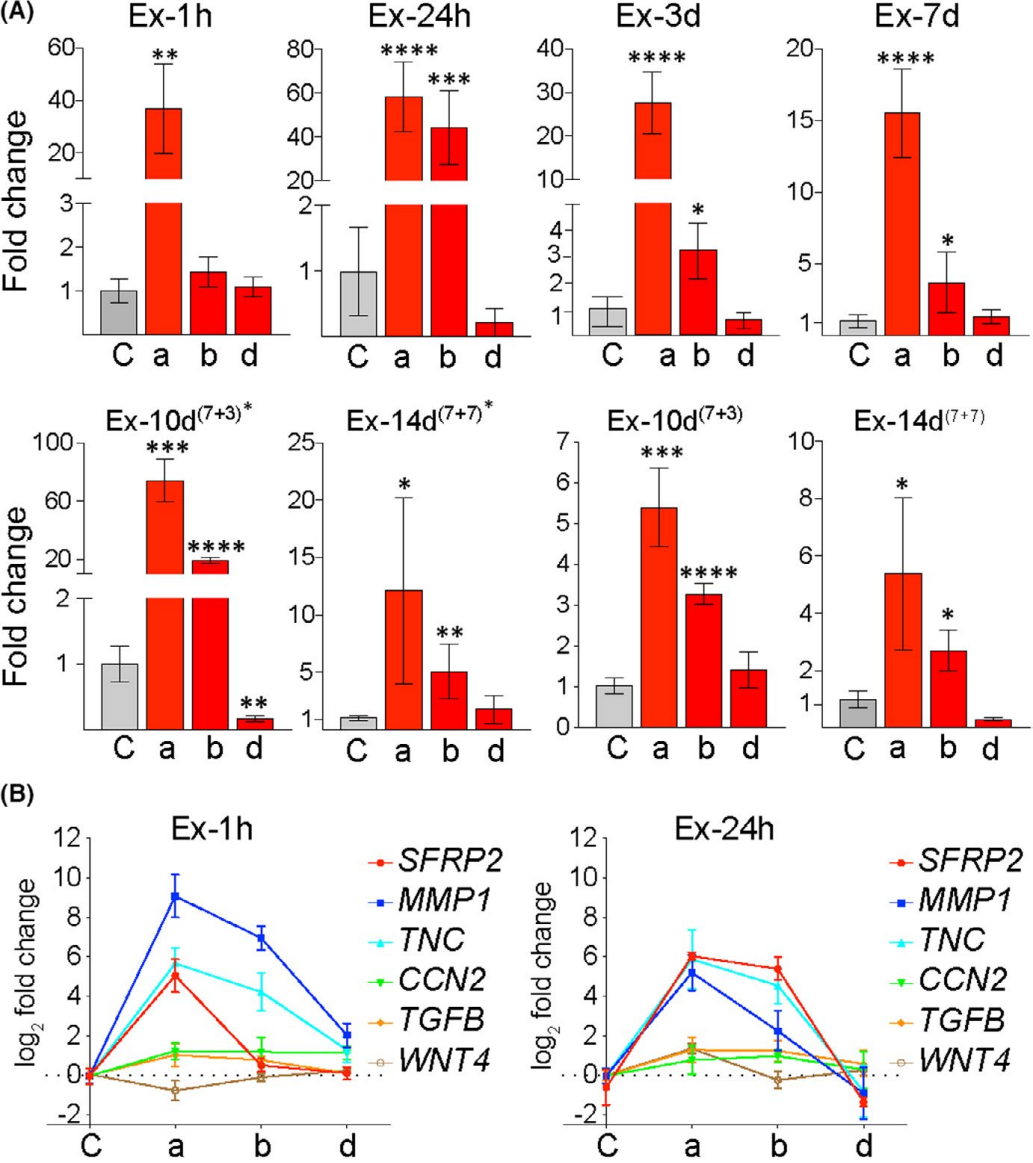
Mechanically induced *SFRP2* expression correlates with a magnitude of stretch and presents similar pattern to known genes involved in mechanotransduction. (A) Quantitative analysis of *SFRP2* expression mapped from one hour to 14 days of TE in skin biopsies harvested from the apex (‘a’), the middle (‘b’) and the periphery (‘d’) of expanded skin. The relative expression was assessed by qRT‐PCR and compared to contralateral unexpanded controls. The values were normalized to *B2M* expression and are presented as the averages for at least three biopsies. Error bars represent SD. Statistical significance are shown as *p≤ 0.05, **p ≤ 0.01, ***p ≤ 0.001, ****p ≤ 0.0001. (B) Gene expression pattern of *SFRP2* and known mechanosensitive genes after one hour (Ex‐1h) and 24 h (Ex‐24h) of expansion

Our most recent RNA‐seq analysis revealed *SFRP2* among the top of the differentially expressed genes in TE. Therefore, we evaluated *SFRP2* transcription levels in skin samples collected at eight timepoints of expansion, from three locations on the expanded skin, by qRT‐PCR. Subcutaneously placed expanders were filled with saline to induce acute stretch for one hour (Ex‐1h), 24 h (Ex‐24h), 3 days (Ex‐3d) and 7 days (Ex‐7d). A second experimental group received two injections of saline over either 10 days or 14 days (Figure S1a,b). The full‐thickness skin biopsies were harvested from the apex (‘a’), middle (‘b’) and peripheral (‘d’) sites of the expanded skin (Figure S1c). Similar locations on the contralateral side served as controls (‘C’). Comprehensive examination of *SFRP2* transcription in all collected biopsies confirmed a correlation between the biopsy position/magnitude of stretch and transcriptional upregulation (Figure 1A). The highest increase in *SFRP2* transcription was detected at the apex of the expanded skin (‘a’), where the highest stretching force was applied. The fold change in the ‘a’ biopsies over the control level was as follows: 37 ± 17 (*p *= 0.0057), 59 ± 16 (*p *< 0.0001), 28 ± 7 (*p *= 0.001) and 16 ± 3 (*p *< 0.0001) for Ex‐1h, Ex‐24h, Ex‐3d and Ex‐7d model, respectively. Type ‘d’ biopsies, collected from the periphery of the expanded skin, revealed no statistically significant changes (Figure 1A). Henceforth, apex samples were examined in this study unless specified otherwise.

To parallel clinical practice, in which subcutaneous expanders are refilled weekly to sustain stretching, we continued TE in four models for a total expansion time of 10 or 14 days. These expanders were filled twice, 7 days apart, two models with 30 ml and two with 60 ml each time. Again, the highest *SFRP2* activation was observed in the ‘a’ biopsies, FC = 74 ± 15 (*p *= 0.0003) for Ex‐10d^(7+3)*^, corresponding to injections of 30 ml at 10 days and 3 days before the tissue was harvested (Figure 1A). Much smaller upregulation, FC = 2.5 ± 0.1 (*p *= 0.0005), for Ex‐10d^(7+3)^ was observed when the expander was filled twice with 60 ml. Similar results were obtained for double‐filled 14‐day TE models: transcription increased by FC = 12 ± 8 (*p *= 0.0149) in Ex‐14d^(7+7)*^ (twice 30 ml) and by 5 ± 3 (*p *= 0.0469) in Ex‐14d^(7+7)^ (twice 60 ml). These results suggest that in prolonged and repeated stretching, smaller volume/lower stress favours higher transcriptional activation of *SFRP2*.

Next, we compared *SFRP2* expression with the expression pattern of known mechanosensitive genes such as *TNC* (Tenascin‐C), *CCN2* (Connective Tissue Growth Factor 2), *MMP1* (Matrix Metallopeptidase 1), *TGFB1* (Transforming Growth Factor Beta 1) and *WNT4* (Wnt Family Member 4).^24^, ^25^, ^26^, ^27^, ^28^ As presented in Figure 1B, among genes included in this analysis, *SFRP2* was one of the three most activated genes in the apical biopsies (‘a’) in both Ex‐1h and Ex‐24h models, and its expression presented a similar pattern to other tested genes. This similarity suggests that *SFRP2* may also be a mechanosensitive gene.

### 3.2 Langerhans cells are a source of SFRP2 in the epidermis

**FIGURE 2:**
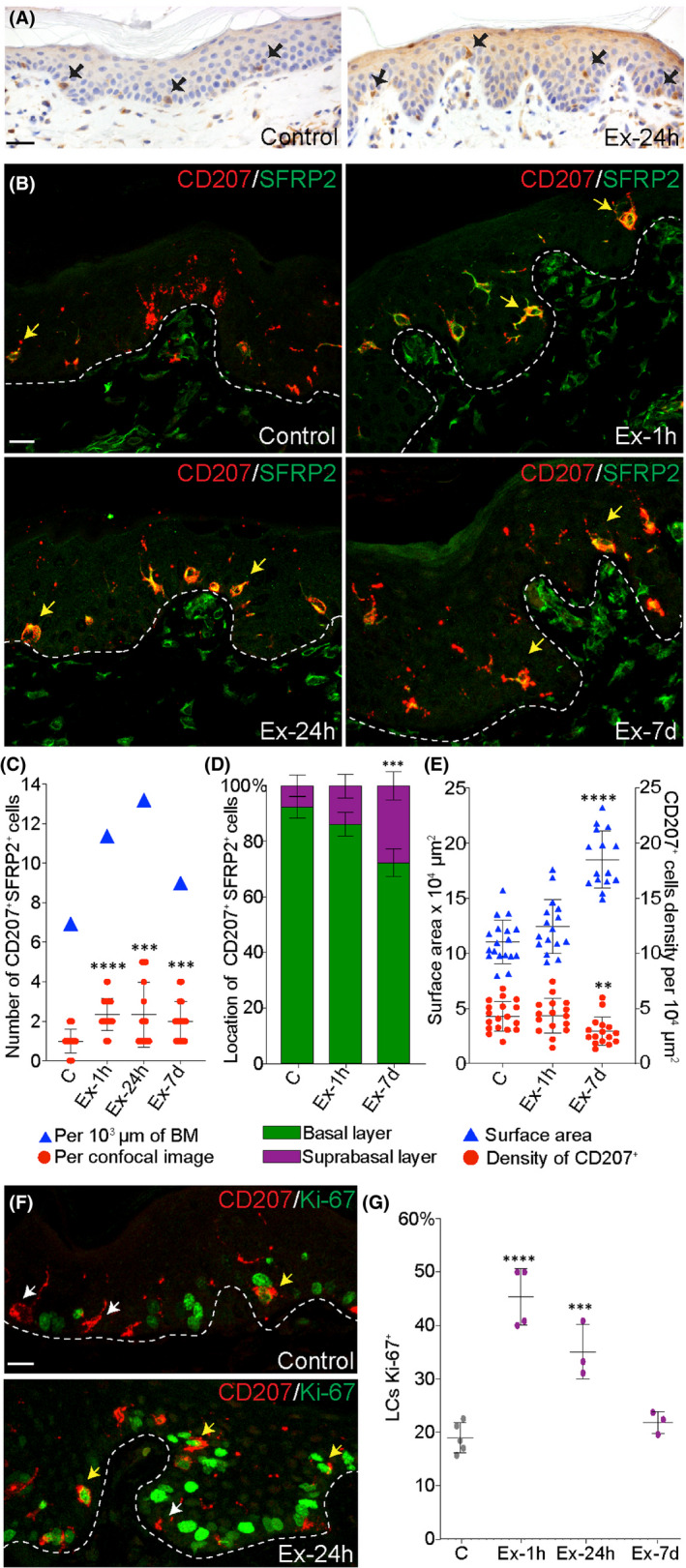
During tissue expansion activated Langerhans cells secrete SFRP2 to epidermal layers of skin through elaborated network of dendrites. Representative images of (A) immunohistochemistry staining of SFRP2 (brown stain) and (B) double immunofluorescent staining of CD207 and SFRP2 in control and expanded skin samples harvested at one hour (Ex‐1h), 24 h (Ex‐24h) and 7 days (Ex‐7d) of expansion. Black arrows in (A) and yellow arrows in (B) indicate SFRP2^+^ and CD207^+^SFRP2^+^ cells, respectively. In (A) slides were counterstained with haematoxylin to mark nuclei (blue stain). (C) Quantitative analysis of double immunofluorescent staining of CD207 and SFRP2. The values are presented as an average number of CD207^+^SFRP2^+^ cells per image (red dots) and per length of basement membrane (BM; blue triangle). (D) Analysis of CD207^+^SFRP2^+^ cells location in the epidermis (n ≥ 10 images per group) in control and expanded skin harvested at one hour (Ex‐1h) and 7 days (Ex‐7d) of expansion. (E) Correlation between surface area of the epidermis and density of LCs during tissue expansion at one hour (Ex‐1h) and 7 days (Ex‐7d) of expansion (n ≥ 10 images per group). (F) Representative images and (G) quantitative analysis of CD207 and Ki‐67 immunofluorescent staining in control and expanded skin samples harvested at one hour (Ex‐1h), 24 h (Ex‐24h) and 7 days (Ex‐7d) of expansion. The yellow and white arrows indicate LCs positive for Ki‐67 and negative for Ki‐67, respectively. The values are presented as an average number of CD207^+^Ki‐67^+^ cells per section (n ≥ 3 per group). Dashed white dashed lines represent epidermal‐dermal junction. Magnification 40×, scale bars indicate 10 µm. Error bars represent SD in (C), (E), (G) and SEM in (D). Significant differences are shown as **p ≤ 0.01, ***p ≤ 0.001, ****p ≤ 0.0001

To explore the role of SFRP2 in TE, we first examined the localization of SFRP2^+^ cells by immunohistochemistry staining (IHC), which revealed evenly distributed SFRP2^+^ cells, morphologically similar to dendritic cells, along the epidermis (Figure 2A). SFRP2^+^, likely dendritic, cells were further examined using double immunofluorescence staining (IF) with an antibody against CD207 (Langerin)^21^ (Figure 2B). The co‐localization of these proteins identified LCs (CD207^+^) as the main source of SFRP2. SFRP2 and CD207 proteins were visible along the LCs dendrites in both control and expanded skin sections, which indicates that SFRP2 is also expressed by LCs in steady‐state conditions. A small number of melanocytes were also positive for SFRP2, as identified by the residual presence of melanin on tissue sections. In addition, we noticed the presence of SFRP2 in dermal fibroblasts, as previously described.^29^

To examine if LCs secrete SFRP2 in human epidermis, we performed analogous double IF staining on normal human skin samples (Figure S1d). Similar to porcine skin, we observed expression and co‐localization of CD207 and SFRP2 on human skin samples in dendritic‐like cells. This result shows molecular similarity between porcine and human skin and indicates potential to apply the findings presented in this report to clinical settings.

### 3.3 Tissue expansion stimulates Langerhans cell proliferation and migration

To better characterize CD207^+^SFRP2^+^ cells, we calculated their quantity and location under normal and experimental conditions. We found that the number of CD207^+^SFRP2^+^ cells, calculated per image, was two times higher at 1 hour (Ex‐1h, *p *< 0.0001), 24 h (Ex‐24h, *p *= 0.0008) and 7 days of TE (Ex‐7d, *p *= 0.0003) (Figure 2C). The increase in the number of CD207^+^SFRP2^+^ cells was also observed after counts were normalized to the basement membrane length, indicating that TE induces secretion of SFRP2 by LCs (Figure 2C).

CD207^+^SFRP2^+^ cells also gradually migrated upward during TE, reaching statistically significant difference in the number of cells located in basal and suprabasal layers by the 7th day (Figure 2D). To further examine the network of LCs in the epidermis, we calculated CD207^+^ cell density and compared it with epidermal thickness, defined as surface area of the epidermis on histological cross‐sections. This showed that at the 7^th^ day of TE, the thickness of the epidermis increased by 68% ± 9% (*p *< 0.0001), and LC density was reduced by 32% ± 14% (*p *= 0.0041), suggesting that the network of LCs was not fully rebuilt by the end of the first week of stretching (Figure 2E).

Given that more CD207^+^SFRP2^+^ cells were detected in expanded skin, we performed double IF staining for CD207 and Ki‐67 to assess if mechanical forces stimulate LC proliferation. Quantitative analysis revealed that the number of CD207^+^Ki‐67^+^ positive cells increased twofold at Ex‐1h (FC = 2.4 ± 0.3, *p *< 0.0001) and Ex‐24h (FC = 1.9 ± 0.3, *p *= 0.0011), and normalized at the 7th day of TE (Figure 2F,G). The increased proliferation of LCs and the migratory shift upward indicates that LCs reorganize themselves, presumably restoring a steady network within the rapidly growing epidermis stimulated by TE.

### 3.4 SFRP2 augments the maturation of keratinocytes

**FIGURE 3:**
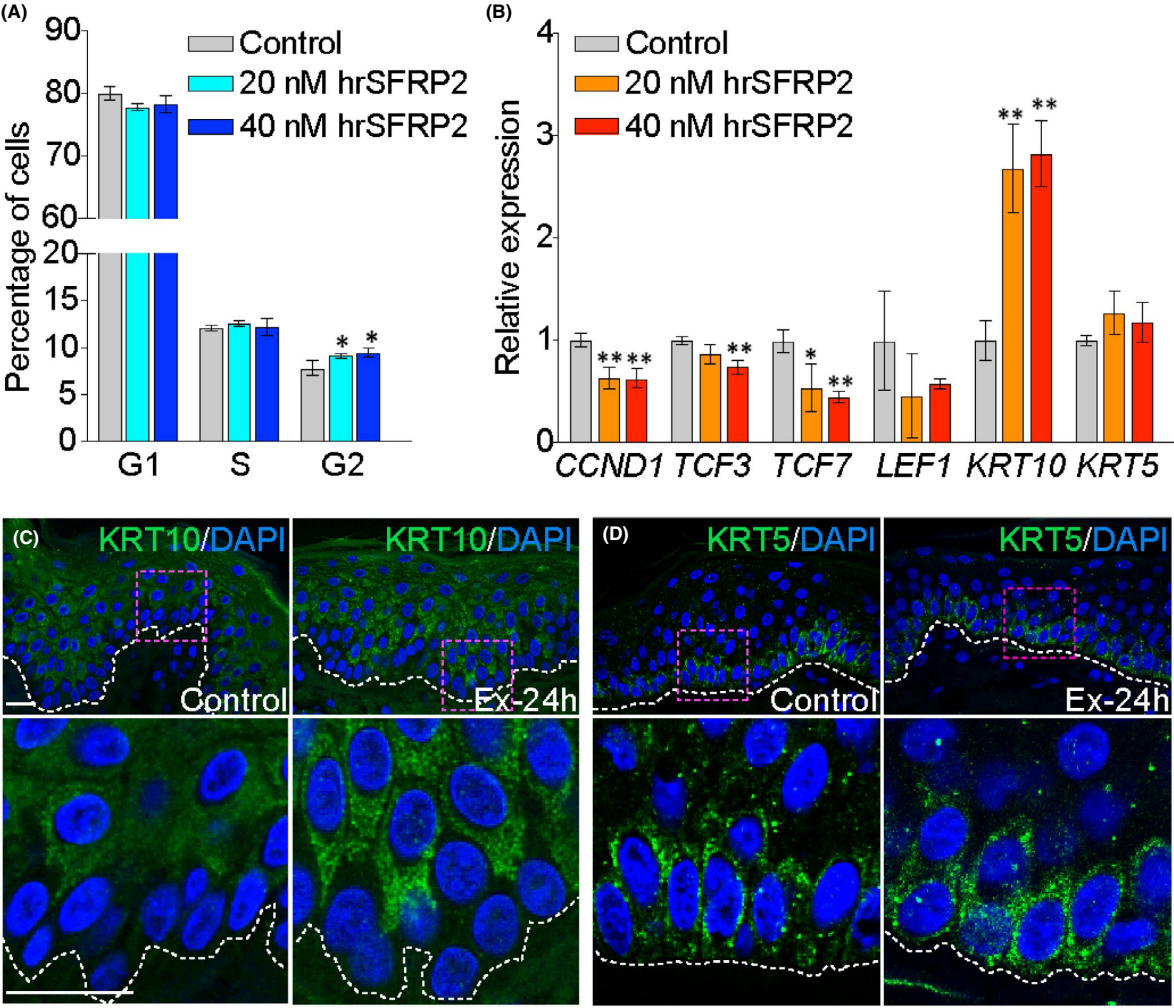
Tissue expansion stimulates keratinocytes maturation through increased expression of SFRP2. (A) The cell cycle distribution analysis on human keratinocytes (HaCaT cells) treated with hrSFRP2 (n = 3). (B) The expression analysis of Wnt target genes and keratins (*KRT5* and *KRT10*) after in vitro treatment with hrSFRP2 on HaCaT cells (n = 3). In (B) the values were normalized to the geometric mean calculated for three references genes: *GAPDH*, *HMBS*, *HPRT1*. Significant differences are shown as *p ≤ 0.05, **p ≤ 0.01, ***p ≤ 0.001, ****p ≤ 0.0001. Error bars represent SD. (C, D) Representative images of immunofluorescent staining of (C) KRT10 and (d) KRT5. Bottom panel, which presents the magnified views of the area marked in the top panel, shows regional increase in KRT10 expression but no significant change in KRT5 expression in the expanded skin. The white dashed lines represent epidermal‐dermal junction. Magnification 40×, scale bar indicates 10 µm

To further investigate the effect of SFRP2 on KCs, we used a confluent culture of human KCs (HaCaT cells) as an in vitro model of normal unperturbed epidermis.^30^ The SFRP2 recombinant protein (rhSFRP2) was delivered for 6 hours in two different concentrations, either 20 nM or 40 nM, to HaCaT cell culture. Testing for cell cycle progression, we found a slightly extended G2 phase by 17% ± 0.5% (*p *= 0.0481) and by 20% ± 1% (*p* = 0.0388), for each respective hrSFRP2 concentration as compared to control, consistent with cell growth and differentiation (Figure 3A).

The transition of basal precursors into the suprabasal layer is associated with a transcriptional switch from *KRT5* to *KRT10*,^31^ resulting in cell cycle withdraw and commitment to terminal differentiation. The examination of *KRT10* and *KRT5* transcription showed an increase in *KRT10* expression by 2.7 ± 0.4 times (*p *= 0.0035) and 2.8 ± 0.3 times (*p *= 0.0011) after treatment with 20 nM and 40 nM of rhSFRP2, respectively, and no significant changes in *KRT5* expression, indicating augmented cell maturation (Figure 3B). However, hrSFRP2 treatment of the subconfluent culture, representing proliferating KCs in otherwise similar conditions, had no significant effect on cell cycle progression and the expression of *KRT5* or *KRT10* (Figure S2a,b). Altogether, this suggests that although SFRP2 can stimulate differentiation, it is not the sole stimulator of KC proliferation.

Next, we examined the distribution of KRT10 and KRT5 *in vivo*, using IF on paraffin sections (Figure 3C,D). KRT10 showed regional increases in the suprabasal layer at 24 hours of TE, but the presence of KRT5 was not different among controls and TE samples. These results suggest that mechanical stimulation increased the commitment of basal precursors towards terminal differentiation. Therefore, it seems probable that TE‐stimulated expression of *SFRP2* augments the early steps of KC maturation *in vivo*.

### 3.5 SFRP2 inhibits Wnt signalling in in vitro culture of keratinocytes

Since SFRP2 is well recognized as a modifier of Wnt signalling, we evaluated the transcription of Wnt target and effector genes, including *CCND1* (*Cyclin D1*) *TCF3* (Transcription Factor 3), *TCF7* (Transcription Factor 7) and *LEF1* (Lymphoid Enhancer Binding Factor 1), using the cell culture conditions described above. The transcription of tested genes was reduced in a dose‐dependent manner (Figure 3B). Specifically, *CCND1* was reduced by 36% ± 6% (*p *= 0.007) and 37% ± 2% (*p *= 0.0051), *TCF3* by 13% ± 1% (*p *= 0.0814) and 26% ± 2% (*p *= 0.0042), *TCF7* by 46% ± 17% (*p *= 0.0387) and 56% ± 8% (*p *= 0.0016) for 20 nM and 40 nM, respectively, without significant change in *LEF1* transcription. These data indicate the inhibitory effect of rhSFRP2 on Wnt signalling.

### 3.6 Tissue expansion affects the subcellular distribution of beta‐catenin

**FIGURE 4:**
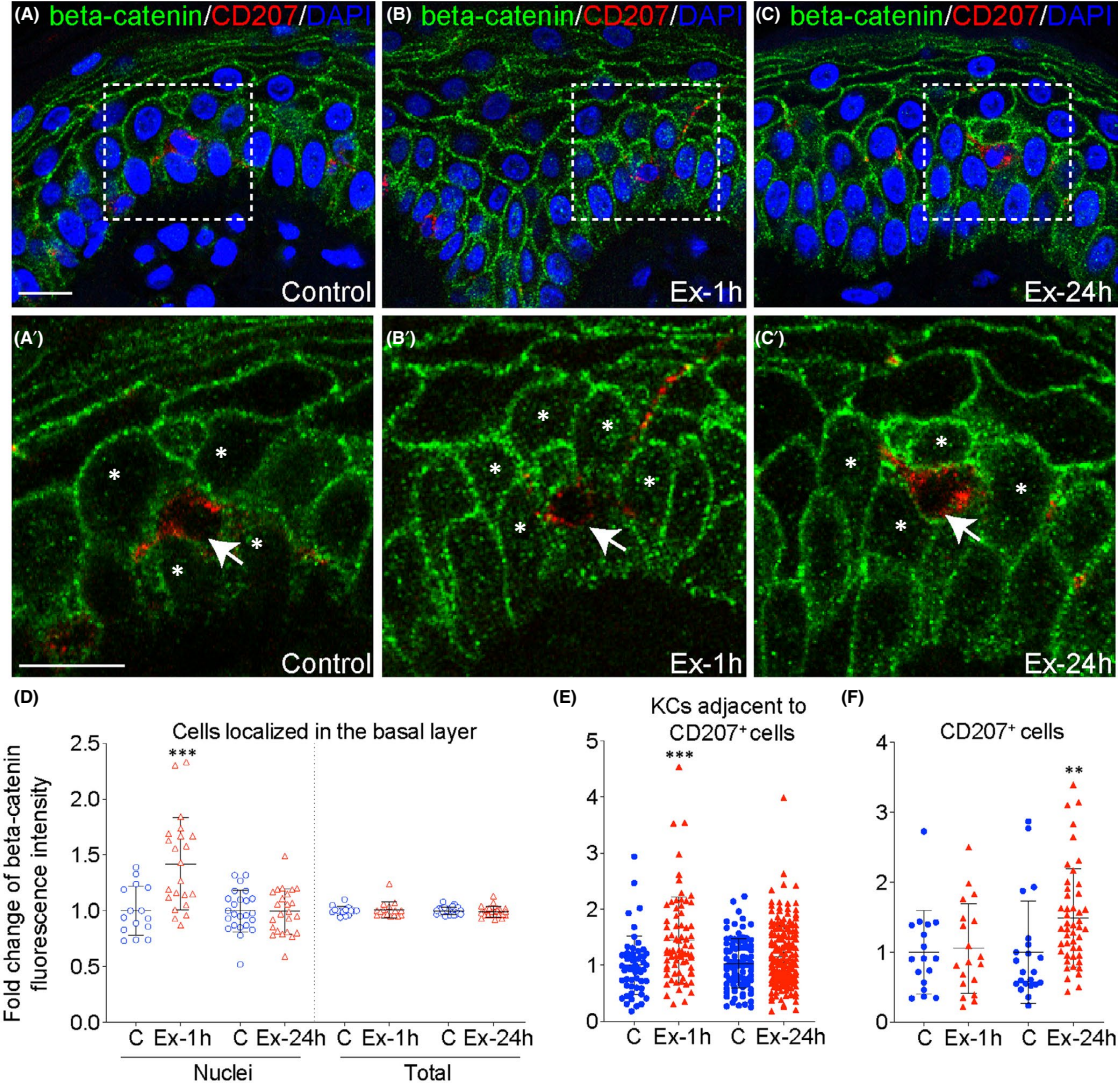
Tissue expansion induces beta‐catenin translocation to nuclei of basal keratinocytes and Langerhans cells. (A–C′) Double immunofluorescent staining of beta‐catenin and CD207 counterstained with DAPI to mark nuclei. In panel (A′–C′) are the magnified views of the area marked by white squares in (A–C). The arrows and asterisks indicate CD207^+^ cells and keratinocytes adjacent to CD207^+^, respectively. Magnification 40×, scale bar indicates 10 µm. (D–F) Quantitative analysis of beta‐catenin intensity in nuclei of (D) basal keratinocytes (n ≥ 15 images), (E) keratinocytes adjacent to CD207^+^ cells (n ≥ 60 cells), and (F) CD207^+^ cells (n ≥ 17 cells). Each dot represents average value for image in (D) and single cells in (E, F). Values represent fold changes between controls and tested samples, and the average value for control was set as one. Significant differences are shown as **p ≤ 0.01, ***p ≤ 0.001. Error bars represent SD

Cells’ ability to respond to mechanical forces by activating specific signalling pathways allows them to adapt to changing environments. To investigate the effect of TE on Wnt/beta‐catenin signalling activation in KCs and LCs, we evaluated cellular localization of beta‐catenin in skin biopsies after one hour (Ex‐1h) and 24 h (Ex‐24h) of expansion (Figure 4A‐F). We observed similar increase by 42%–45% (*p *≤ 0.0002) in nuclear beta‐catenin localization in basal KCs (42% ± 12%, *p *= 0.0001) and in KCs adjacent to LCs (CD207^+^ cells; 45±24%, *p*=0.0002) after one hour of expansion (Ex‐1h) (Figure 4A‐B′,D,E). Interestingly, the nuclear accumulation of beta‐catenin in these cells declined to control level by 24 h of expansion (Figure 4A‐E). These results reveal that b‐catenin translocation to the nuclei of KCs is stimulated directly by stretch and not by the proximity of LCs. Accordingly, we predict that the force inflicted by TE initiates signalling of beta‐catenin, which translocates to the nuclei of KCs and induces KC proliferation.

By contrast, beta‐catenin measured in the nuclei of LCs was unchanged at one hour, but increased by 49% ± 23% (*p *= 0.0094) at 24 h of TE (Figure 4F). The total amount of beta‐catenin in basal KCs and LCs was not changed, confirming that the protein had been translocated. These results show that TE induces activation of Wnt/beta‐catenin pathway exclusively in basal KCs and LCs. The temporal shift in nuclear accumulation of beta‐catenin in the nuclei of these cells reflects a difference in their mechanosensitivity.

## 4 DISCUSSION

Our study aimed to better understand the molecular effects of mechanical stretching on the multilayered epidermis. Structurally and functionally, porcine skin is considered very similar to human's and more suitable for experimental models of TE than ‘loose‐skinned’ rodents.^32^ Porcine and human epidermis, unlike mouse, possess rete ridges which are highly impacted by expansion.^7^

We found that mechanical stretching, inflicted by TE, upregulates *SFRP2* expression, and its transcription level strongly correlates with the magnitude of stretch, corresponding to the location of biopsies. We discovered that LCs proliferate and migrate in response to stretch, and secrete SFRP2 by elongated dendrites to different layers throughout the epidermis. We also assert that SFRP2 may be a mechanosensitive gene as its expression pattern in TE is similar to genes known to respond to mechanical forces. Our studies suggest that SFRP2 augments KC maturation rather than proliferation, which is controlled instead by Wnt/beta‐catenin.

The observed upregulation of *SFRP2* expression in expanded skin is likely caused directly by mechanical stretch. The literature supports this assertion by showing that mechanical forces can directly stretch chromatin and induce gene transcription.^33^ However, a positive correlation between the relatively highest *SFRP2* transcription level (Figure 1A) and nuclear accumulation of beta‐catenin in LCs (Figure 4F) also suggests that *SFRP2* may act downstream of Wnt signalling. There are similarities in the response to strain between bone and skin cells, which support this assumption. For example, mechanical force is known to increase the expression of Wnt/beta‐catenin target genes, including *Sfrp1* and *Ccnd1*, in osteoblasts and osteoclasts.^34^, ^35^, ^36^ Sfrp2 was also identified as the most upregulated extracellular antagonist of Wnt signalling in a Splotch‐delayed mouse embryo model of bone and joint development.^28^ Additionally, in our study, the activation of *SFRP2* in expanded skin closely resembles the transcriptional dynamics of known mechanosensitive genes such as *TNC* and *MMP1*.^24^, ^25^, ^26^, ^27^, ^28^ In conclusion, the transcription of *SFRP2*, activated either by chromatin stretching or Wnt/b‐catenin, is induced by mechanical stimuli with a similar pattern to mechanosensitive genes. Furthermore, as expansion is prolonged, SFRP2 expression declines as skin adapts to stretch by growing, attempting to restore homeostasis.

Moreover, we discovered that LCs are the main source of SFRP2 in the epidermis and that stretch stimulates their proliferation and migration from the basal to suprabasal layer, presumably to restore the homeostatic network. This resembles LC behaviour during wound healing.^37^ LCs are a unique cell type; based on their ontology, they belong to tissue‐resident macrophages, but they function as dendritic cells programmed by interactions with the microenvironment. Signals, such as cytokines, chemokines, pathogens and UV radiation, can induce transcriptional changes in LCs,^38^ and their proliferation.^19^, ^39^ The decrease in LCs density in the epidermis at 7 days of expansion suggests that mechanically stimulated excessive epidermal growth, driven mostly by KC proliferation, results in temporal disruption in LCs network and homeostasis. Consequently, observed in this study, increased proliferation and migration of LCs, and SFRP2 secretion are driven by mechanical forces likely in an attempt to adapt to homeostatic disruption caused by skin expansion.

We observed that after one our of expansion, Wnt signalling is exclusively activated in basal KCs, preceding the stretched‐induced peak in KC proliferation.^8^ Our results are in agreement with data showing that quiescent epithelial cells exposed to mechanical strain re‐enter the cell cycle through activation of the beta‐catenin pathway.^40^ Numerous studied indicate that Wnt/beta‐catenin signalling controls proliferation of basal precursors in the epidermis.^41^, ^42^, ^43^ Loss of beta‐catenin in the interfollicular epidermis (IFE) results in reduced basal cell proliferation and epidermal thinning, and conversely, transient activation of epidermal beta‐catenin leads to increased IFE thickness.^41^, ^44^ In the plasma membrane, beta‐catenin is bound to E‐cadherin and alpha‐catenin complex through adherens junctions, which are involved in mechanotransduction.^45^ Importantly, it has been shown that the E‐cadherin/beta‐catenin complex at the adherence junction can be opened by physiological strain *in vivo*, which allows a fraction of beta‐catenin to be released and translocate to the nucleus to activate transcription.^46^ Several other studies identified beta‐catenin as an effector of mechanical signals.^47^, ^48^ Our data from in vivo and in vitro studies suggest that increased *SFRP2* expression in expanded skin might prevent excessive activation of Wnt/beta‐catenin pathway in basal KCs. These results are in agreement with other papers reporting SFRP2 as a Wnt pathway antagonist.^49^, ^50^, ^51^

Interestingly, at 24 h of expansion, Wnt signalling was no longer upregulated in basal KCs, but nuclear beta‐catenin was now increased in LCs. LCs are firmly tethered to KCs through E‐cadherin, which is complexed with beta‐catenin, located at adherens junction,^52^ and this connection is important for LC migration and maturation.^53^ It was also shown that beta‐catenin is a positive regulator of LC differentiation in response to TGF‐beta‐1^54^ and breaking the E‐cadherin bond on dendritic cells leads to the activation of beta‐catenin/TCF/LEF dependent transcription.^55^ Therefore, the increase in beta‐catenin accumulation in LCs nuclei indicate temporal disruption in the connection between KCs and LCs, and explains the observed increase in LCs migratory activity under mechanical forces.

Based on in vitro treatment of KCs with hrSFRP2 showing increased KRT10 expression, a marker of KC differentiation^31^ and cell cycle progression, we predict that a role of SFRP2 in TE is to stimulate KC differentiation. Consistent with the current understanding of Wnt signalling's role in IFE,^41^ KCs which encounter more SFRP2, resulting in less Wnt activity, would progress towards differentiation as shown by the increased expression of KRT10 in vitro and *in vivo*. A previous study even showed that SFRP2 can inhibit KC proliferation in longer treatment.^56^

In conclusion, we have outlined a mechanism through which LCs and SFRP2 act as regulators of epidermal homeostasis in TE. Their mechanically stimulated activation prevents overstimulation of the Wnt/beta‐catenin signalling pathway. Although TE typically takes several weeks to generate enough skin for surgical reconstruction, this study indicates that stretching skin initiates molecular responses in as little as one hour. A better understanding of the initiating components of skin growth can contribute to creating skin pre‐treatments to stimulate faster growth and regeneration, even in compromised tissue. Our investigation of LCs and SFRP2 highlights their therapeutic potential in this effort.

## 5 CONCLUSIONS

In conclusion, our study provides previously unreported evidence that mechanical forces can stimulate SFRP2 secretion by LCs to promote skin growth and restore epidermal homeostasis. This effect is achieved by regulation of Wnt/b‐catenin signalling, resulting in augmentation of KC maturation. Altogether, our work uncovers a new biological role for SFRP2 and LCs in mechanically induced skin growth and regeneration, and helps us understand the molecular response of skin during TE.

## CONFLICT OF INTEREST

The authors have no conflict of interest to declare. This work was prepared while Jacek Topczewski and Jolanta M. Topczewska were employed at Lurie Children's Hospital of Chicago and Northwestern University. The opinions expressed in this article are the author's own and do not reflect the view of the National Institutes of Health, the Department of Health and Human Services, or the United States government.

## AUTHOR CONTRIBUTIONS

**Joanna K. Ledwon:** Conceptualization (equal); Data curation (lead); Formal analysis (lead); Investigation (lead); Methodology (lead); Visualization (lead); Writing – original draft (equal). **Elbert E. Vaca:** Investigation (supporting); Writing – review & editing (supporting). **Chiang C. Huang:** Resources (equal); Writing – review & editing (supporting). **Lauren J Kelsey:** Investigation (supporting); Writing – review & editing (equal). **Jennifer L. McGrath:** Investigation (supporting); Writing – review & editing (supporting). **Jacek Topczewski:** Resources (equal); Writing – review & editing (supporting). **Arun K. Gosain:** Conceptualization (supporting); Funding acquisition (lead); Supervision (equal); Writing – review & editing (supporting). **Jolanta M. Topczewska:** Conceptualization (equal); Formal analysis (supporting); Funding acquisition (supporting); Investigation (supporting); Methodology (equal); Supervision (lead); Visualization (supporting); Writing – original draft (lead).

## Supporting information

Fig S1

Fig S2

Supplementary Material

## Data Availability

All data generated or analysed during this study are included in this article and its supplementary information files.
