# Supplementary figures and images for: Langerhans cells and SFRP2/Wnt/beta‐catenin signalling control adaptation of skin epidermis to mechanical stretching
Source: J Cell Mol Med. 2022 Jan 12;26(3):764–75. doi: 10.1111/jcmm.17111 (PMC8817127; doi:10.1111/jcmm.17111)

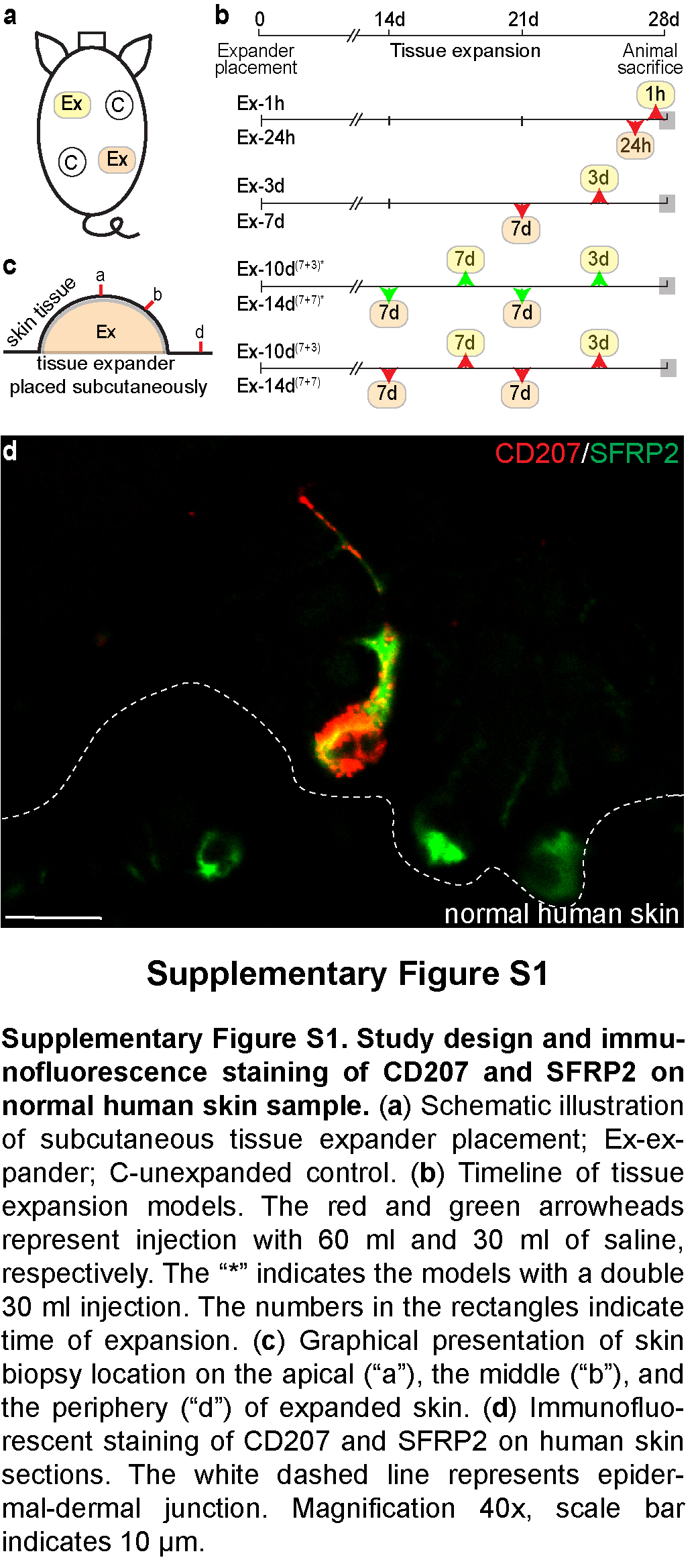

Supplement: Supplementary file 1 — Fig S1 [file JCMM-26-764-s002.jpg]

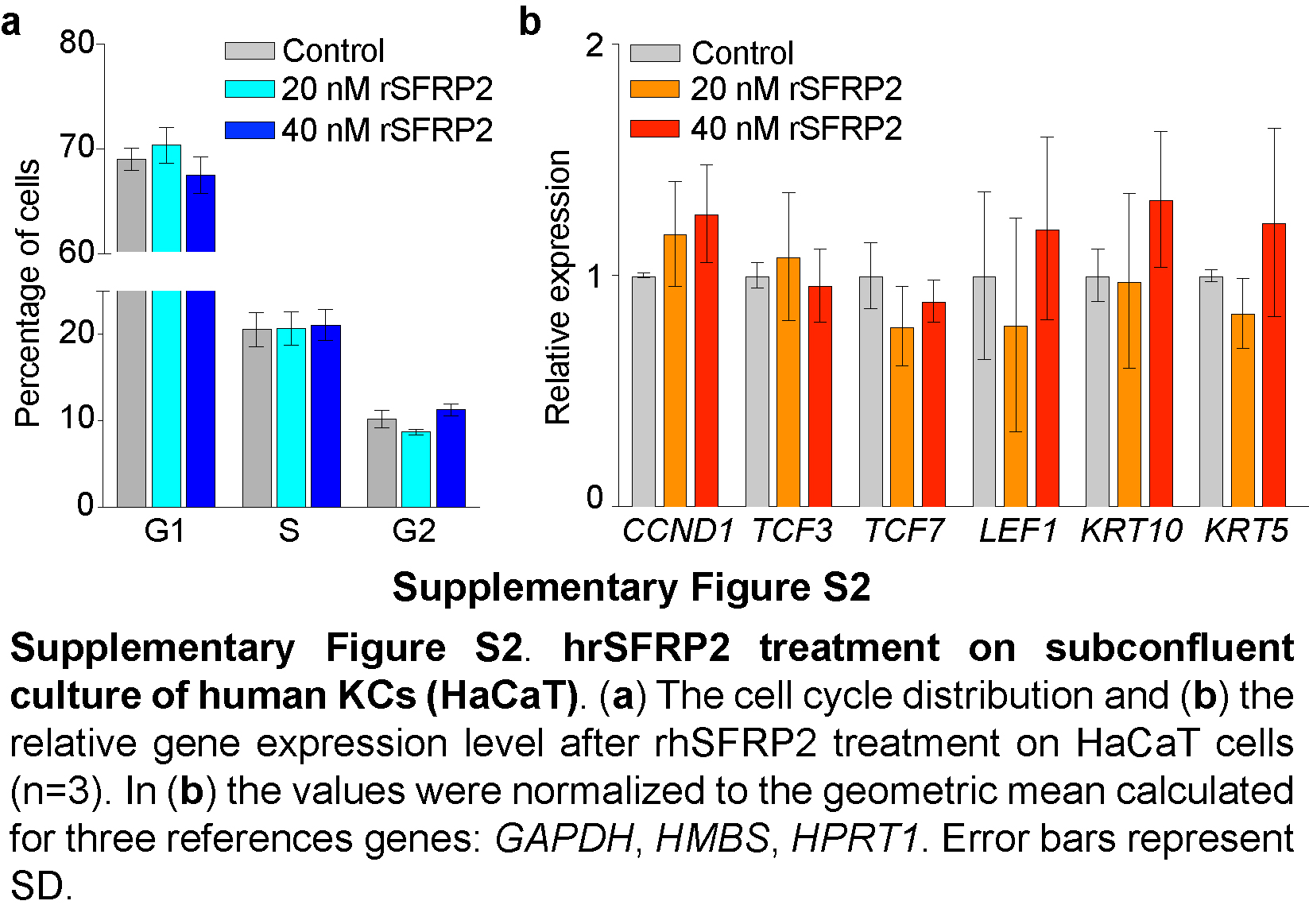

Supplement: Supplementary file 2 — Fig S2 [file JCMM-26-764-s001.jpg]
