# Supplementary material for: Langerhans cells and SFRP2/Wnt/beta‐catenin signalling control adaptation of skin epidermis to mechanical stretching
Source: J Cell Mol Med. 2022 Jan 12;26(3):764–75. doi: 10.1111/jcmm.17111 (PMC8817127; doi:10.1111/jcmm.17111)
Supplement: Supplementary file 3 — Supplementary Material [file JCMM-26-764-s003.docx]

**Supplementary Material**

**Supplementary Figure S1.** Study design and immunofluorescence staining of CD207 and SFRP2 on normal human skin samples.

**Supplementary Figure S2.** An *in vito* hrSFRP2 treatment on subconfluent culture of human keratinocytes.
